# Public Health Graduates' Perceptions of the Educational Environment Measured by the DREEM

**DOI:** 10.3389/fpubh.2022.738098

**Published:** 2022-03-09

**Authors:** Fang-Rong Xu, Yang Yang

**Affiliations:** ^1^Department of Clinical Nutrition, Affiliated Jinhua Hospital, Zhejiang University School of Medicine, Jinhua, China; ^2^Department of Prevention and Health Care, Affiliated Jinhua Hospital, Zhejiang University School of Medicine, Jinhua, China

**Keywords:** educational environment, graduates, public health, DREEM, score

## Abstract

**Objective:**

Students' perceptions of the educational environment have a significant impact on their behavior and academic progress. This study aims to measure medical graduates' perception of the educational environment within the School of Public Health at Wuhan University in China.

**Methods:**

The survey was conducted by emails sent to 119 graduates, and 93 valid questionnaires were returned. The DREEM was used to assess the medical graduates' satisfaction with the educational environment.

**Results:**

The average score on the scale was 126.02 (±18.27). The scoring rate of the areas ranged between 61.06 and 67.11%. The area with the highest score was “perception of teachers.” The area with the lowest score was “academic self-perception.” No difference was found between genders. Except for “perception of atmosphere,” the total scores and other areas showed differences in graduation time.

**Conclusion:**

The educational environment at the School of Public Health at Wuhan University was satisfactory. The program contributed to the graduates' later careers. The information obtained in this study identified some areas for improvement.

## Introduction

The educational environment includes the campus environment and the subjective and objective factors related to school, teaching, and learning. In teaching and learning, the subjective states of students and their interpersonal relationships are also included (1). Some research shows the educational environment has an impact on the students' experience and output of learning. Students' perceptions of the educational environment have a significant impact on their behavior and academic progress, affecting students' impression of the school and their academic success. To put it another way, students' satisfaction with the educational environment stimulates them to enjoy learning and realize the goal of medical education (2–4). The World Federation for Medical Education considers the educational environment is one of the areas that should be assessed while evaluating medical education programs (5).

Evaluations of the educational environment emerged as early as the 1960s. There are many tools used to assess the educational environment (6–9). The Dundee Ready Education Environment Measure (DREEM) is the most widely used and recognized one. This scale is used to measure the educational environment under reform (10). It is also used to evaluate the effectiveness of university curriculum and education programs (5, 11). In China, the DREEM is translated by the education center of Chinese Medical Sciences University. It has been used in several medical schools and demonstrated high validity and reliability (12).

Although widely used in medical education, few studies in the literature have adopted this measure in public health education. This study investigates the educational environment by using the DREEM through the public health graduates' feedback. By comparing a number of factors and exploring problems in the educational environment of public health, we hope to identify problems in education and provide a better environment for teaching and academic studies.

## Materials and Methods

### Study Design and Sample

A descriptive cross-sectional design was employed for this study. This study was undertaken by graduates from the School of Public Health at Wuhan University, Wuhan, China. A total of 119 graduates from 2008 to 2013 of the School of Public Health at Wuhan University were eligible to participate in the study. Those graduating in 2012 and 2013 were considered students in the later stage of reform, and the other students were representative of the early stage. This study was approved by the School of Public Health and supported by the Global Health Institute of Wuhan University. Wuhan University is one of the first universities to carry out a long-term program cultivating integrated international talents with practical abilities.

### Data Collection

A total of 119 graduates were sent an email with a questionnaire inviting them to participate in the study, and those who rejected the study did not respond to the email. The DREEM was used to assess the medical graduates' satisfaction with the educational environment. If more than 3 questions of DREEM were not answered, the questionnaire was considered invalid. If there are <2 questions are not be answered, the question would be assigned average scores of each question. Basic demographic information, like sex and year of graduation, was collected. If “gender” or “year of graduation” are not available and unable to add, the questionnaire was also considered invalid.

### Instruments

The DREEM contains 50 items measuring five dimensions of the educational environment: perception of learning (item 1, 7, 13, 16, 20, 22, 24, 25, 38, 44, 47, 48), perception of teaching (item 2, 6, 8, 9, 18, 29, 32, 37, 39, 40, 50), academic self-perception (item 5, 10, 21, 26, 27, 31, 41, 45), perception of atmosphere (item 11, 12, 17, 23, 30, 33, 34, 35, 36, 42, 43, 49), and social self-perception (item 3, 4, 14, 15, 19, 28, 46).

The DREEM consists of 50 items evaluated on a scale from 0 (strongly disagree) to 4 (strongly agree). Reverse coding was required for items 4, 8, 9, 17, 25, 35, 39, 48, and 50, and they were evaluated on a scale from 0 (strongly agree) to 4 (strongly disagree). Thus, the total score of the DREEM was 200. According to the total scores, the educational environment was divided into four levels; 0–50: very poor environment; 51–100: plenty of problems; 101–150: more positive than negative; 151–200: excellent environment. The reliability and validity of DREEM was approved in previous studies (13, 14).

### Data Analysis

EpiData 3.1 was used to set up a database, and Statistical Product and Service Solutions (SPSS) software (version 20.0, SPSS Inc., Chicago, IL) was employed to conduct statistical analyses (15). The main statistical methods are descriptive statistics and two independent sample *t*-tests. The significance level was 0.05.

## Results

### Questionnaires Collected

This investigation excluded questionnaires failing to provide contact information and those with a flat refusal. One hundred nineteen questionnaires were administered, and 93 questionnaires were collected. The return rate was 78.15%.

Among the respondents, 41 participants graduated from 2008 to 2011 (44.4%), and 52 graduated from 2012 to 2013 (55.9%). Fifty-four participants were male (58.1%), and 39 were female (41.9%) (Table 1).

**Table 1:** Comparison of DREEM score between sex and reform.

	**Perception of learning**	**Perception of teachers**	**Academic self-perception**	**Perception of atmosphere**	**Social self-perception**	**Total**
Total (*n* = 93)	29.34	29.53	19.54	30.10	17.35	126.02
Maximum score	48	44	32	48	28	200
Percentage of maximum score (%)	61.13	67.11	61.06	62.71	61.96	63.01
Female (*n* = 39)	30.74 ± 5.41	30.03 ± 4.78	19.82 ± 3.41	31.00 ± 4.81	17.85 ± 2.81	129.69 ± 17.74
Male (*n* = 54)	28.33 ± 6.07	29.17 ± 4.45	19.33 ± 3.81	29.44 ± 5.16	17.00 ± 2.79	123.37 ± 18.35
The value of *P*	0.051	0.376	0.526	0.144	0.154	0.100
Earlier stage(*n* = 41)	30.98 ± 4.88	31.12 ± 4.14	20.73 ± 3.28	31.05 ± 4.56	18.17 ± 2.76	132.05 ± 15.64
Later stage (*n* = 52)	28.06 ± 6.34	28.27 ± 4.57	18.60 ± 3.65	29.35 ± 5.33	16.71 ± 2.72	121.27 ± 18.92
The value of *P*	0.017^*^	0.002^*^	0.004^*^	0.101	0.012^*^	0.004^*^

* *Differences are statistically significant*.

### Score of the DREEM

The overall score for the DREEM on average was 126.02 ± 18.267. The scoring rate for each subscale was 61.06–67.11%. “Perception of teachers” was the area with the highest score, and “academic self-perception” was the area with the lowest score (Table 1).

The score of items ranged from 1.53 to 3.23. Among them, two items scored below two points: “the teaching is often stimulating” and “cheating is a problem within the program” (Table 2).

**Table 2:** Mean and standard deviation for individual items of the DREEM.

**Item**	**Mean**	**SD**
I am encouraged to participate during lectures	2.74	0.736
The lecturers are knowledgeable	2.81	0.680
There is a good support system for students who get stressed	2.08	0.912
I am too tired to enjoy the program	2.59	0.837
Learning strategies which worked for me before continue to work for me now	2.22	0.919
The clinical faculty espouse a patient centered approach to clinical work	2.60	0.662
The teaching is often stimulating	1.86	0.880
The faculty ridicule the students	2.99	0.814
The faculty are authoritarian	2.75	0.880
I am confident about my passing this year	2.65	0.717
The atmosphere is relaxed during clinical teaching	2.72	0.578
The program is well scheduled	2.70	0.763
The teaching is student centered	2.42	0.851
I am rarely bored with the program	2.17	0.928
I have good friends within the program	3.23	0.420
The teaching helps to develop my competence	2.91	0.564
Cheating is a problem within the program	1.53	1.017
The clinical faculty have good communication skills with clients	2.38	0.736
My social life is good	2.37	0.882
The teaching is well focused	2.56	0.714
I feel I am being well prepared for my profession	2.31	0.859
The teaching helps to develop my confidence	2.47	0.731
The atmosphere is relaxed during lectures	2.63	0.777
The teaching time is put to good use	2.42	0.812
The teaching over emphasizes factual learning	2.05	0.948
Last year's work has been a good preparation for this year's work	2.47	0.916
I am able to memorize all I need	2.09	0.893
I seldom feel lonely	2.46	0.760
The faculty are good at providing feedback to students	2.66	0.667
There are opportunities for me to develop interpersonal skills	2.63	0.749
I have learned a lot about empathy in my profession	2.48	0.746
The faculty provide constructive criticism here	2.60	0.724
I feel comfortable in lectures socially	2.88	0.657
The atmosphere is relaxed in lectures or rounds	2.73	0.645
I find the experience disappointing	2.42	0.901
I am able to concentrate well	2.78	0.568
The faculty give clear examples	2.72	0.665
I am clear about the learning objectives of the classes	2.68	0.678
The faculty get angry in lecture	2.81	0.824
Faculty are well prepared for their teaching sessions	2.45	0.730
My problem solving skills are well developed here	2.67	0.648
The enjoyment outweighs the stress of the program	2.32	0.849
The atmosphere motivates me as a learner	2.32	0.899
The teaching encourages me to be an active learner	2.37	0.918
Much of what I have to learn seems relevant to a career within veterinary medicine	2.66	0.715
My accommodation is pleasant	2.46	0.891
Long term learning is emphasized over short term learning	2.91	0.789
The teaching is too faculty centered	2.11	0.949
I feel able to ask the questions I want	2.42	0.771
The students irritate the faculty	2.76	0.826

Except for “perception of atmosphere,” graduates of the earlier stage scored higher than the graduates of the later stage in the other four areas, and the differences were statistically significant (*p* < 0.05) (Table 1).

Out of the 50 items, there were significant differences in 14 items between graduates before and after reform. They were “perception of learning” (7, 22, 44), “perception of teaching” (6, 18, 29, 32, 37), “academic self-perception” (5, 21, 27), “perception of atmosphere” (23), and “social self-perception” (3, 28) (Table 3). Among them, the item with the largest proportion of differences was the area of “academic self-perception.”

**Table 3:** Individual items scores have statistic difference between groups.

**Item**	**Earlier stage**	**Later stage**	***p***
There is a good support system for students who get stressed	2.39 ± 0.67	1.83 ± 1.00	0.020
Learning strategies which worked for me before continue to work for me now	2.44 ± 0.90	2.04 ± 0.91	0.036
The clinical faculty espouse a patient centered approach to clinical work	2.76 ± 0.54	2.48 ± 0.73	0.039
The teaching is often stimulating	2.12 ± 0.75	1.65 ± 0.93	0.010
The clinical faculty have good communication skills with clients	2.61 ± 0.70	2.19 ± 0.72	0.006
I feel I am being well prepared for my profession	2.66 ± 0.66	2.04 ± 0.91	<0.001
The teaching helps to develop my confidence	2.66 ± 0.69	2.33 ± 0.73	0.029
The atmosphere is relaxed during lectures	2.90 ± 0.44	2.42 ± 0.92	0.001
I am able to memorize all I need	2.32 ± 0.91	1.90 ± 0.85	0.026
I seldom feel lonely	2.71 ± 0.72	2.27 ± 0.75	0.005
The faculty are good at providing feedback to students	2.85 ± 0.48	2.50 ± 0.75	0.007
The faculty provide constructive criticism here	2.78 ± 0.65	2.46 ± 0.75	0.034
The faculty give clear examples	2.95 ± 0.59	2.54 ± 0.67	0.002
The teaching encourages me to be an active learner	2.59 ± 0.74	2.19 ± 1.01	0.033

## Discussion

### Overall Scores

A good educational environment can help students obtain the necessary abilities for their further careers and benefits their individual development. It allows students to keep physically and mentally healthy. In this study, total DREEM mean score was 126.02 ± 18.267 with percentage of maximum score was 63.01% (Table 1), which indicates overall positive educational environment perception than negative. The range of total scores on the DREEM scale of students who came from developed countries, such as Sweden, Germany, Britain, and the United States, was 120–150 (16–22) while the range of less developed countries' students was 105–125 (11, 13, 23, 24). The total scores of the DREEM scale of students from China were about 120 (25–27). It demonstrated the educational environment of the School of Public Health of Wuhan University was more positive than negative on the whole. Students are generally satisfied with the educational environment. There is still much room for improvement compared with developed countries.

### Scores of Areas

According to the total scale score, students' perception of all subscales was more positive than negative, but further improvements are still needed (Table 1).

Compared with students in other countries, those in Germany, Britain, Sweden, and other developed countries had the highest scoring rate in two areas, “students' perception of the environment” and “students' social interaction and self-perception” (11, 12, 18, 20, 21), since the economies of those countries are highly developed and their school environments and facilities are better than other countries. In addition, those countries gave importance to all aspects of training besides study. Therefore, students' interpersonal skills and self-confidence were stronger than students in other countries. There were also more ways to achieve goals.

Less developed countries, such as Malaysia and Sri Lanka, had the highest scoring rate in “students' perception of learning” and “students' perception of academics” (11, 24). Students in these countries have monotonous lives at school and most of their time is spent on study. They are satisfied with their studies and academics. But in a less developed economy, the hardware facilities and learning environments were not satisfying. Also, there was only one way to achieve personal goals.

Students at Chinese universities gave the highest-scoring rates on the subscale of “students' perception of learning” and “students' perception of teachers” (25–27), which might be related to the traditional Chinese concepts that teachers and their teaching should be respected. The scores were lowest in “students' perception of academic” and “students' social interaction and self-perception.” Similar to students from less developed countries, most students in China lack the opportunity to develop comprehensive abilities, especially during undergraduate education. Medical students mainly rely on textbook knowledge with fewer opportunities to participate in social activities or research groups. For this reason, the School of Public Health at Wuhan University has been committed to exploring a more advanced educational model of cultivating students with comprehensive quality.

### Scores of Items

According to the DREEM inventory researchers' advice, items with an average score of ≤ 2.0 requires attention (19, 28). In this study, two items' average scores were ≤ 2.0. One of them was “The teaching is often stimulating” (1.86) (Table 2). This was similar to other studies (29). It might be because the teaching content was relatively outdated and failed to keep up with the new era. The teaching effect was not good because of the dull teaching methods. The other item was “Cheating is a problem within the program” (1.53), which was reported in another study (18). It had the lowest score of the 50 items. Because of opportunistic behavior by some students, examinations did not fairly reflect students' learning outcomes, which reflected the importance of comprehensive talent cultivation and evaluation. Above all, these results indicated that the reform of medical education is imperative.

### Comparison of Scores Between Groups

The total scores and scores in different subscales between sexes were not all significant (Table 1), which was similar to some research (11, 16, 18, 23). However, the scores were statistically different between males and females in other research (5, 6).

Except for the subscale of “students' perception of learning,” graduates of the earlier stage scored higher than graduates of the later stage in total and subscale scores. The graduates of the earlier stage experienced more benefits of reform due to accumulated work experience. They applied knowledge and skills to work more adequately and achieve work objectives better; thus, their perception of the educational environment during the academic year was better.

### Strength and Limitations

At present, few studies have focused on evaluating the educational environment of public health. Studies aimed at public health education reform are rare. Under the continuous threat of SARS, Ebola, and the novel coronavirus, the cultivation of public health talent is crucial. The exploration at the School of Public Health at Wuhan University put theories into practice and had some success. There were several limitations of this study. For example, the sample size was small as this study was conducted at one university, and only 93 students participated. The subjects were all graduates, and their memories might have some bias due to the length of time since graduation and social experience.

## Conclusion

The study into the graduates' perception of the educational environment of the School of Public Health of Wuhan University indicated that it was satisfactory overall. The program identified that cultivating integrated international talents with practical abilities contributes to a graduate's later career. Some shortcomings do exist in the educational environment, and further reform is needed to strengthen it.

## Data Availability Statement

The original contributions presented in the study are included in the article/supplementary material, further inquiries can be directed to the corresponding author.

## Ethics Statement

An Ethics Committee favourable opinion was not required. All data sets used were completely anonymous and there was no risk of disclosure of personal data.

## Author Contributions

F-RX contribute, to compilation of literature, sending questionnaires, and reviewing the manuscript. YY edited the questionnaire, collected and analyzed the data, and was a major contributor in writing the manuscript. Both authors read and approved the final manuscript.

## Conflict of Interest

The authors declare that the research was conducted in the absence of any commercial or financial relationships that could be construed as a potential conflict of interest.

## Publisher's Note

All claims expressed in this article are solely those of the authors and do not necessarily represent those of their affiliated organizations, or those of the publisher, the editors and the reviewers. Any product that may be evaluated in this article, or claim that may be made by its manufacturer, is not guaranteed or endorsed by the publisher.
